# Immediate and Long-Term Pull-Out Bond Strength of 3D-Printed Provisional Crowns

**DOI:** 10.1155/2024/7205011

**Published:** 2024-09-04

**Authors:** Joyce R. C. dos S. Siqueira, Rita M. M. Rodriguez, Nathalia de C. Ramos, Marco A. Bottino, João P. M. Tribst

**Affiliations:** ^1^ Department of Dental Materials and Prosthodontics São Paulo State University (UNESP) Institute of Science and Technology, São José dos Campos 12245-000, Brazil; ^2^ Department of Reconstructive Oral Care Academic Centre for Dentistry Amsterdam (ACTA) 1081 LA, Amsterdam, Netherlands

## Abstract

**Background:** Over the past decade, 3D printing technology has revolutionized various fields, including dentistry. Provisional restorations play a crucial role in prosthetic rehabilitation, necessitating the evaluation of their bond strength with different provisional cement agents.

**Aims:** This study is aimed at assessing the immediate and long-term bond strength of 3D-printed dental crowns using three provisional cement agents.

**Materials and Methods:** Provisional crowns (*N* = 36) were manufactured using 3D modeling software and cemented in dentin analogues (G10 Nema resin). After the crowns' fabrication, they were randomly divided into three groups (*n* = 12) for cementation with Relyx Temp 3M ESPE, Provicol—VOCO, and Meron—VOCO. Tensile strength tests were conducted using a universal testing machine, with half of the specimens subjected to 2000 thermal cycles before testing. Finite element analysis was employed to assess tensile stress distribution.

**Results:** Statistical analysis (two-way ANOVA and Tukey's test at a 95% confidence level) revealed significant effects of cement type (*p* = 0.006) and thermal aging (*p* = 0.001) on bond strength. Glass ionomer cement exhibited the highest immediate resistance, while all types of cement were adversely affected by thermal aging, resulting in decreased bond strength.

**Conclusion:** Thermal aging significantly alters the properties of 3D printing resin and affects the bond strength of provisional cement with 3D-printed crowns. Despite the adverse effects of thermal aging, glass ionomer cement demonstrated the highest immediate resistance. Clinicians should carefully consider these findings when selecting provisional cements for 3D-printed crowns.

## 1. Introduction

Over the past decade, fields such as medicine, pharmaceuticals, and bioengineering have adopted three-dimensional (3D) printing for a range of applications [1]. However, dentistry has been among the most prominent beneficiaries of 3D printing. From surgical guides to personalized prostheses, the technology has streamlined various aspects of dental treatment and has been employed in various applications, including the manufacturing of surgical guides, orthodontics, endodontics, and maxillofacial surgery [2]. New technologies have been developed for the production of models, prostheses, implants, fixed and removable prosthetic appliances, aligners, and mostly provisional restorations [3, 4].

Regarding provisional restorations, a multitude of materials and techniques have been utilized over time. Initially, acrylic resins and polymethylmethacrylate (PMMA) were common options but had limitations such as distortion, porosity, and lack of adequate fit [5]. New materials, like CAD/CAM blocks and 3D printing resins, have emerged as more effective and versatile alternatives. Recent studies have shown the strength and adaptability of these materials, suggesting significant advancements in provisional restorations [6].

The selection of provisional materials should consider specific clinical needs and material characteristics [5, 7]. Different fabrication techniques, including direct and indirect manufacturing, can be employed based on clinical circumstances and professional preferences. Regardless of the manufacturing method, provisional restorations are crucial in prosthetic rehabilitation, providing immediate protection, function, and stability while aiding in diagnostic assessment before definitive procedures [8]. They must meet specific requirements, including good marginal adaptation, proper fit, retention, and resistance to displacement during chewing. Additionally, they should be durable, nonirritating, exhibit low heat generation, and be aesthetically acceptable. Ease of removal and cementation, along with low cost and a low incidence of allergic reactions, are also essential considerations [5–8].

Regarding clinical usage, provisional restorations are typically intended for short-term use, generally spanning 1–2 weeks, and are often produced chairside due to the need for immediate application [9]. Consequently, the use of 3D printing for these restorations is less common, as it involves additional time and effort that may not be justified for such a brief period of use. However, for patients undergoing extensive or full mouth rehabilitation, the scenario changes significantly. In these cases, a “medium to long-term” provisionalization is often required, extending the duration of provisional crown usage and thereby justifying the utilization of 3D printing technology [10]. This advanced manufacturing method can provide more precise and customized restorations, enhancing the overall treatment outcome for these patients [8–10].

After production, provisional restorations should be provisionally cemented. Provisional cementation is the process of fixing indirect restorations using interim cementing agents. These agents serve various purposes, including periodontal tissue evaluation, hygiene assessment, and ensuring proper masticatory function and occlusion [9]. They also aid in promoting effective recovery for the soft tissue before the placement of a definitive restoration. Their primary function is to seal the provisional restoration to the prepared tooth, preventing marginal infiltration and pulp injury while also allowing for easy removal [9, 11]. In addition, provisional cements do not have adhesion/bonding to the tooth structure, and their retention depends heavily on macroretention forms.

Commonly used provisional materials include calcium hydroxide, zinc oxide and eugenol, glass ionomer, and eugenol-free options [12, 13]. It is worth noting that there is a lack of studies regarding the provisional cementation of 3D-printed restorations. Therefore, the present study is aimed at evaluating immediate and long-term bond strength with three different provisional cement agents on the retention of 3D-printed dental crowns. The null hypothesis is that there is no significant difference in the immediate and long-term bond strength among the three different provisional cement agents on the retention of 3D-printed dental crowns.

## 2. Materials and Methods

In addition to in vitro strength tests, this study employs finite element analysis (FEA) to visualize the tensile stress distribution within the cement layer. This dual approach allows for a detailed examination of how different cements perform under stress and helps to identify potential weaknesses not apparent through experimental testing alone.

### 2.1. Elastic Modulus Assessment

FEA will be used to accurately measure the tensile stress during the pull-out test of provisional crowns. However, it is necessary to first characterize the mechanical properties of the 3D printing resin used in the present study. To do so, a specimen was designed in Rhinoceros software Version 6.0 (McNeel North America, Seattle, United States). The design was exported in STL format to the printer software to establish printing patterns. Thus, three specimens were printed in a rectangular shape with dimensions of 60 × 10 × 5 mm [14]. After printing, the specimens were immersed in isopropyl alcohol and placed in an ultrasonic bath for thorough cleaning. After drying on absorbent paper, they were placed in a light polymerizing unit for final polymerization for 1 h.

After 24 h, the elastic modulus of the materials was determined using pulse receiver equipment (MOD 5900 PR, Olympus, United States) connected to an oscilloscope (TDS 1002, Tektronix, United States) using the pulse-echo method. To calculate the elastic modulus, it was necessary to determine the density of the specimens, which was measured using the Archimedes method on a precision balance. After measuring the dry mass of the specimens, the test was applied.

### 2.2. Crown Manufacturing

**Figure 1:**
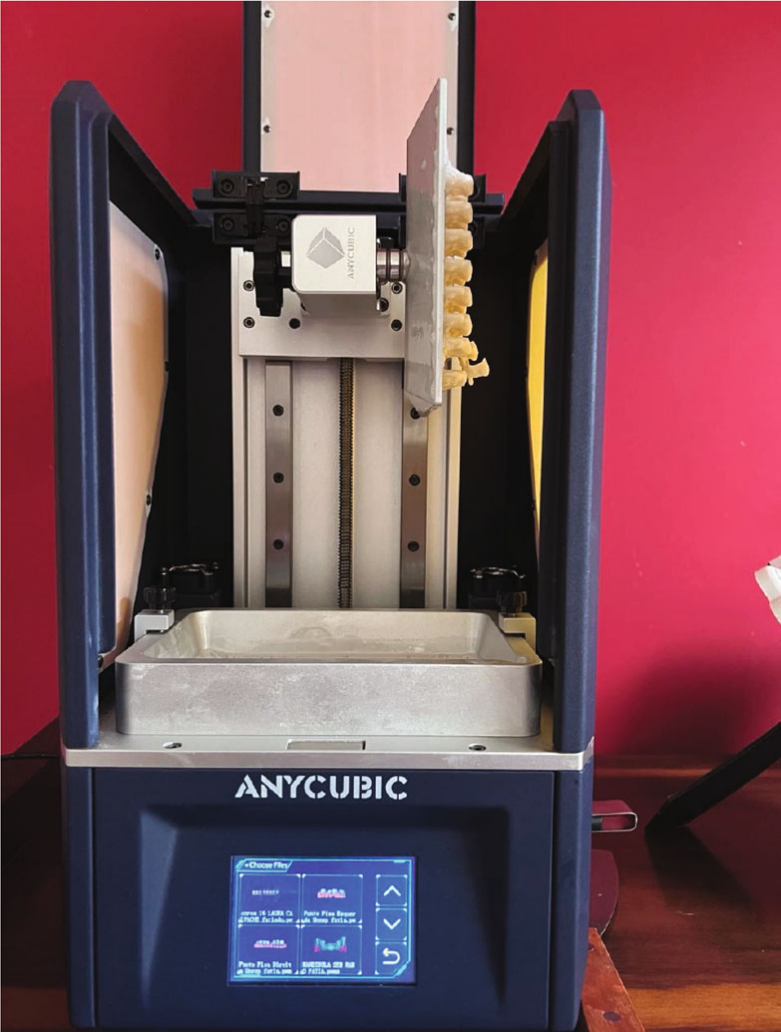
3D printer with the finished set of provisional crowns.

**Figure 2:**
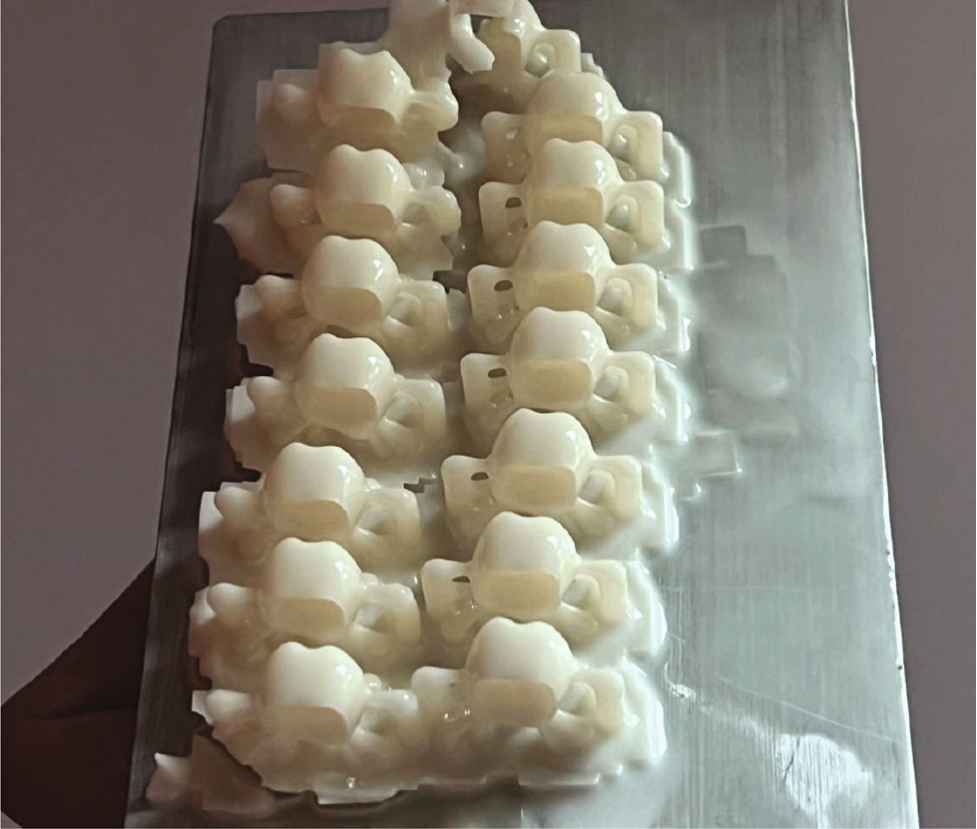
3D printed set of provisional crowns.

For the evaluation of tensile strength, a preparation for the maxillary first molar was designed in 3D modeling software (Rhinoceros 6.0) and machined in dentin analogues (G10 Nema resin) resin, a valid dentin analog material for bond strength testing [15]. After obtaining the preparation, it was scanned using a desktop scanner (Cerec InLab MCXL, Sirona Dental Systems, Bensheim, Hessen, Germany), and a crown design for the maxillary first molar was created. The generated STL file was exported to the printer software, and printing parameters were defined (such as support type, exposure time, and layer thickness). The crowns (Resilab Temp 3D Lot 1582) were printed at a 90° angle relative to the printing bed (Figures 1 and 2).

**Figure 3:**
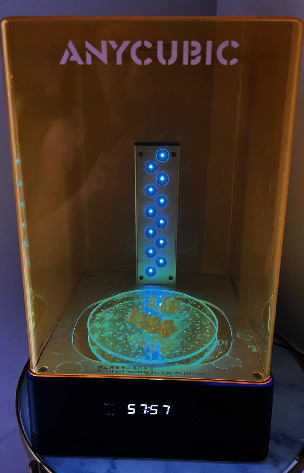
Postprocessing of provisional crowns.

After printing, the crowns were washed in isopropyl alcohol for 4 min, dried on absorbent paper, and polymerized in the Photon Anycubic light polymerizing unit for 1 h without removing the supports (Figure 3). With the fabricated crowns (*n* = 36), they were randomly divided into three groups (*n* = 12) for each provisional cement used (overview of the investigated cement and restorative materials presented in Table 1): Relyx Temp 3M ESPE (zinc oxide without eugenol), Provicol—VOCO (calcium hydroxide), and Meron—VOCO (glass ionomer). The preparations were embedded in PVC tubes with polyurethane, and after 24 h, the crowns were cemented. The cemented assembly was embedded in PVC tubes with acrylic resin and stored in an incubator at 37°C for 24 h.

### 2.3. Aging and Pull-Out Test

**Figure 4:**
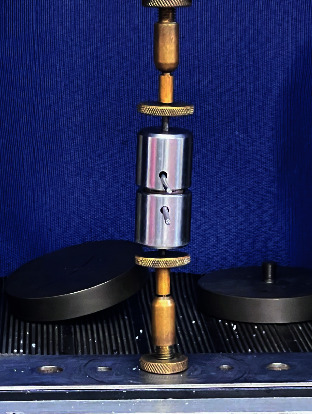
Pull-out testing device during the retention force testing.

**Figure 5:**
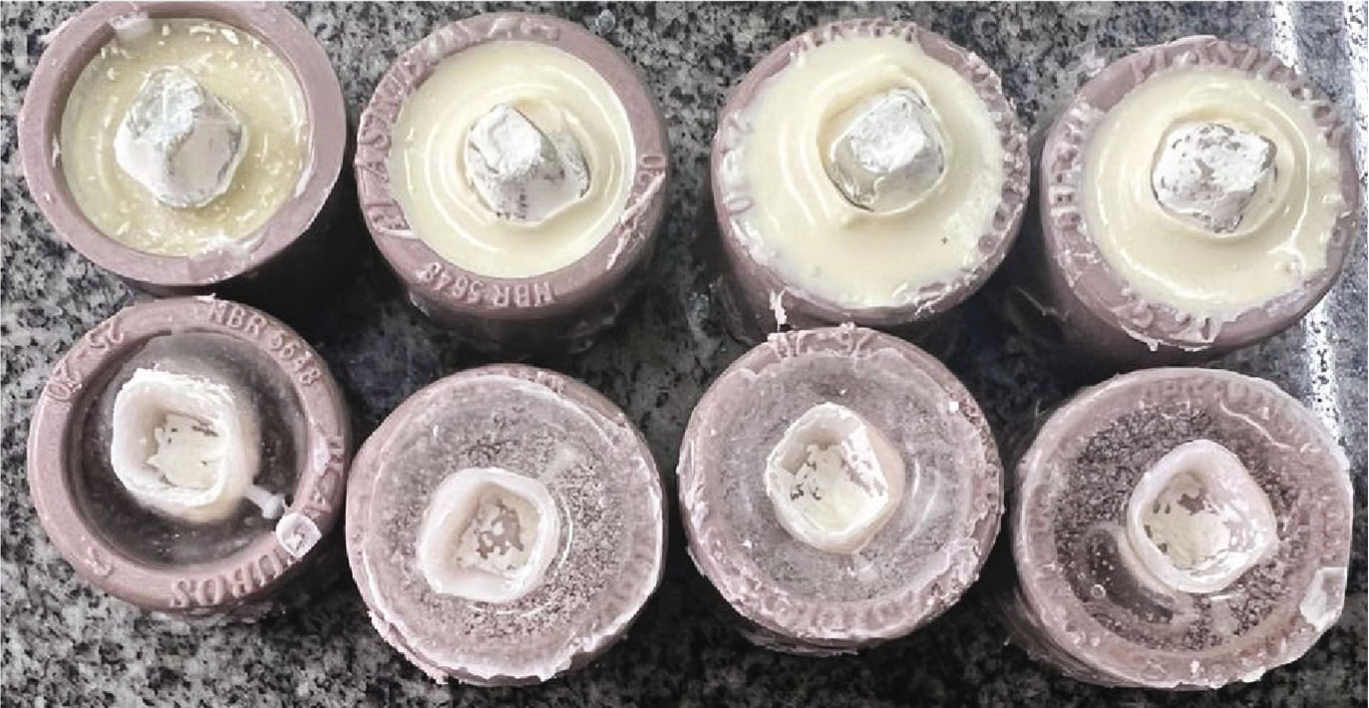
Detached crowns and their respective abutment after the pull-out testing.

After the cement setting, the tensile strength test was conducted on half of the groups (*n* = 18) using a universal testing machine (EMIC DL-1000, Equipamentos e Sistema de Ensaio Ltda., São José dos Pinhais, Paraná, Brazil) with a load cell of 1000 Kgf at a speed of 0.5 mm/min until the detachment of the crown/preparation assembly occurred. The other half of the crowns (*n* = 18) underwent aging in a thermocycler (Biopdi cycler, BioPDI, São Carlos, Brazil) for 2000 cycles, alternating between a 30-s dwelling time in the water at 55°C and a 30-s dwelling time in the water at 5°C. After aging, the specimens were subjected to the tensile test again using the universal testing machine, with the same load cell and speed as described earlier (Figures 4 and 5). The obtained data were used as input in the FEA and statistically analyzed using a two-way ANOVA and Tukey's test at a 95% confidence level.

### 2.4. FEA

The tensile forces measured during the experimental tests were used as input loads for the FEA simulations. A previously validated 3D model of a maxillary first molar was utilized to assess the tensile stress of the cement layer [15]. This model, comprising the crown, cement (100 *μ*m), root, and fixation cylinder, was imported into Rhinoceros CAD software (Version 5.0 SR8, McNeel North America, Seattle, WA). The same details used for in vitro specimen preparation were used in the CAD software to create a NURBS volumetric model.

The volumetric solids were then imported into the analysis software (ANSYS 19.0, ANSYS Inc., Houston, TX) in STEP (STandard for the Exchange of Product model data) format. A mesh convergence test (10%) established 318,184 nodes and 216,455 tetrahedral elements for the finished design.

**Figure 6:**
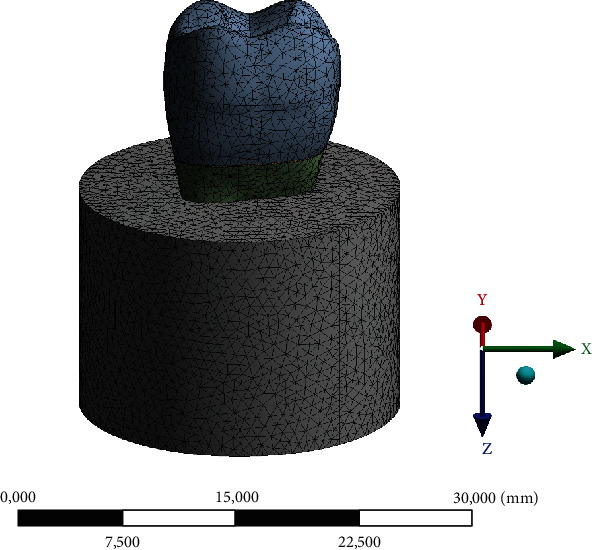
Mesh model for the pull-out simulation.

Perfectly bonded contacts were assumed, and structural mechanical analysis involved loading the groups with the load calculated during the pull-out test (Figure 6). The loading area was distributed at all external areas of the crown, while the base was constrained as per the experimental setup. The material properties simulated in this study are dentin analogue (18.6 GPa, 0.3 *ν*) [15], PMMA for the fixation cylinder (2.2 GPa, 0.3 *ν*) [16], zinc oxide without eugenol cement (4.95 GPa, 0.3 *ν*) [15, 17], calcium hydroxide cement (0.3 GPa, 0.23 *ν*) [18], and glass ionomer cement (GIC) (8 GPa, 0.2 *ν*) [19].

The maximum principal stress (MPS) criterion was selected to analyze tensile stress concentration in the cement layer, with results presented in stress maps and peak values recorded.

## 3. Results

The following results (Table 2) were obtained from the average of the values before and after thermal aging.

The obtained values were statistically evaluated using two-way ANOVA and Tukey's 95% test. The results are observed in Table 3. All factors were statistically significant, both the groups (*p* = 0.006) and the aging (*p* = 0.001).

**Figure 7:**
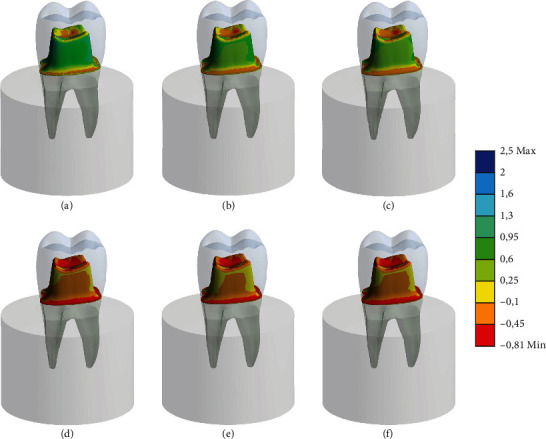
Tensile stress calculated in the cement layer according to the retention force from in vitro testing. (a) IOV immediate, (b) HC immediate, (c) OZnE immediate, (d) IOV after aging, (e) HC after aging, and (f) OZnE after aging.

The results of the FEA indicate that all crowns exhibit homogeneous stress distribution across their surfaces, as depicted in Figure 7. However, there are noticeable differences between the crowns subjected to thermal aging and those that were not. Specifically, the crowns subjected to thermal aging exhibit lower stress values, suggesting a lower strength required to detach them from the preparation compared to immediately tested groups. Furthermore, slight variations in stress distribution are observed among the different provisional cement materials. Among these materials, GIC demonstrates the most promising behavior, displaying relatively high strength levels compared to other cement types. This suggests that GIC may offer improved retention properties when used for provisional cementation of 3D-printed crowns. FEA revealed that GIC exhibited the most uniform stress distribution, which corresponds with its highest immediate bond strength observed experimentally (2.55 MPa). In contrast, the stress concentrations observed in the calcium hydroxide cement align with its lower immediate bond strength (1.81 MPa). These findings suggest that the more uniform stress distribution contributes to higher bond strength.

## 4. Discussion

The choice of a cementing agent should be based on various factors such as the degree of dental retention, duration of provisional prosthesis use, fabrication technique, and pulp vitality. Provisional materials must be biocompatible, have low mechanical resistance, and be easy to handle [5, 7]. In the present study, all cements showed a decrease in the bond strength after aging, suggesting that the crowns would easily detach after a period. This can be associated with the change of mechanical properties from the restorative material, with a reduction of its stiffness and therefore higher flexibility during removal. This is in agreement with previous studies that also showed a negative effect of thermocycling on the properties of 3D printable resin for dental applications [4, 20].

For the tensile strength test, specimens in the shape of a molar were printed, and preparations with the selected anatomical shape were milled in dentin analog (G10 Nema resin) to simulate conditions in the oral cavity. Dentin analogues (G10 Nema resin) possess mechanical properties similar to dentin [21]. This includes factors such as strength and elasticity. In addition, this dentin analogue can be used to simulate bonding with other dental materials such as composite resins or dental cement [22].

The combination of experimental and FEA results provides a more complete understanding of the performance of different cements. The high bond strength of GIC can be attributed to its uniform stress distribution, as demonstrated by the FEA. In addition, it can be observed that the finishing line and occlusal wall are the most predominant regions of the highest stress peaks, suggesting possible outcomes of ether debonding or fracture over time.

The selected cement comprises the most commonly used materials for provisional crown cementation [5]. However, convergence angle [23] and preparation height [24] are additional factors influencing crown retention and thus affecting tensile strength. In this study, the same preparation, with identical characteristics, was used for all groups, with the only variation being the cement used and the aging of the crown.

Based on the test results, it was observed that among the types of cement used, glass ionomer tested 24 h after cementation showed the best performance, followed by zinc oxide without eugenol (Relyx) and calcium hydroxide (Provicol). However, after thermal aging, all tested cements showed similar reduced performance, with no statistical difference between them. The values obtained for glass ionomer cementation are higher than those found in the literature for provisional cementation, indicating its potential for final cementation. A previous investigation used zirconia abutment to cement provisional crowns with different types of cement and then performed the pull-out test [25]. The authors found different pull-off forces in the range between 3.0 and 16.8 N after thermocycling, which according to them allows manual detachment of the restoration by the dentist [25]. In the present study, the higher values can be justified by the use of dentin analogue as preparation instead of zirconia, being a more favorable substrate for high bond strength values than the polycrystalline ceramic.

Another in vitro study investigated the retention forces of an intact bovine incisor and acrylic resin crown (Dencor Classical Dental Articles Ltda) cemented with calcium hydroxide. Testing a modified cementation technique found that pull-out forces for this cement were lower (mean 0.58 MPa) than the conventional technique (mean 1.08 MPa) [5], which were lower than the results with cemented in our study (mean 1.81 MPa) in the immediate group. The present study reported an average value of 1.81 MPa for similar composition cement for the immediate measurement. However, the outcome may differ when different surface treatments are used in the substrate or in the crown itself [26].

In this study, GIC, due to its more fluid characteristic compared to other tested cement, may have initially exhibited better performance, as the cement thickness was thin and homogeneous. In contrast, calcium hydroxide and zinc oxide have a higher viscosity, leading to thicker layers along the crown and preparation. Studies have shown that increased cement thickness directly affects marginal adaptation and retention [27, 28].

It is important to notice that thermocycling involves subjecting dental materials to cycles of temperature changes, simulating the conditions they would experience in the oral cavity. This repeated expansion and contraction can lead to microleakage, where gaps form between the cement and the tooth or restoration [29]. In this scenario, some dental cement may undergo degradation or changes in properties when exposed to thermal cycling.

GIC exhibits characteristics such as biocompatibility, viscosity, and an elastic modulus similar to dentin. However, like other materials, it has certain limitations, including opacity, higher surface roughness, and low mechanical properties. These issues arise from syneresis and imbibition when the cement comes into contact with the oral environment, particularly due to saliva. Imbibition (water uptake) leads to the dissolution of ions, making the cement less resistant and more soluble. This can result in dimensional changes, loss of mechanical properties, and the formation of cracks [30, 31]. Therefore, in the present study, it is likely that the cement experienced both syneresis and imbibition, leading to lower mechanical resistance compared to other cement used after thermal aging. Associated with that, the thermocycling also affected the restorative material, reducing its elastic modulus and probably affecting its bondability [32]. Those factors occur simultaneously and are both responsible for reducing the retention force of provisional crowns.

When testing bond strength, studies often employ microtensile or microshear methods to reduce the defect population in the cement and to facilitate the calculation of bond strength in megapascals using simple geometries that allow for the application of straightforward mathematical formulas [33]. However, in clinical settings, restorations are typically larger than these microsamples. This study used anatomical crowns, closely replicating the clinical setup, and applied FEA to calculate the values in megapascals. This approach allowed for the use of anatomical samples that are more representative of real clinical conditions rather than relying on bars or cylinders. Additionally, the cement application and retention mechanisms in this study closely mimicked those found in actual clinical scenarios, enhancing the relevance and applicability of the findings.

While crown cementation on dentin analogues (G10 Nema resin) has been extensively studied using resin cement, there are limited comparisons between data obtained from human dentin and dentin analogues (G10 Nema resin) when provisional cement is used. This extrapolation of data applies mainly to resin cement and not to cement-like glass ionomer and zinc phosphate [21]. However, the data obtained in this study align with references found in the literature. In addition, all in vitro studies have limitations, and further research using natural teeth and mechanical cycling is suggested.

In addition, the present study acknowledges several limitations in the FEA model utilized. Firstly, the model incorporated isotropic materials, which do not accurately represent the anisotropic nature of biological tissues found in the oral cavity [34]. Additionally, the contacts between structures in the model were considered ideal, failing to account for the more complex and variable interactions that occur in vivo (such as partially debonded regions). The cement layer within the model was uniformly set at a thickness of 100 mm, a simplification that does not reflect the potential variability and inconsistency in clinical scenarios. These idealized conditions differ significantly from the dynamic and heterogeneous environment of the oral cavity [35], and as such, the findings should be carefully extrapolated when considering the mechanical behavior of provisional crowns in actual clinical settings and when compared with long-term restorative materials [36].

## 5. Conclusions

In conclusion, the thermal aging process significantly alters the properties of the 3D printing resin, resulting in a decrease in the elastic modulus following thermocycling. While glass ionomer, calcium hydroxide, and zinc oxide eugenol cement are indicated for provisional cementation of printed crowns, all are adversely affected by thermal aging. Despite this, GIC demonstrates the highest immediate resistance among the tested materials. Therefore, careful consideration should be taken into account when selecting provisional cements for printed crowns.

## Figures and Tables

**Table 1:** Overview of the investigated cements and restorative materials.

**Symbols**	**Product**	**Product's presentation**	**Manufacturers**	**Indication**
IOV	Meron (glass ionomer)	35 g powder 15 mL liquid	VOCO	For cementing of crowns, inlays, and onlays; bridges; pins; and orthodontic bands
OZnE	Relyx Temp 3M ESPE (zinc oxide without eugenol)	25 g base paste 18 g catalyst paste	3M	Cementing temporary restorations, provisional cementation of permanent restorations, and cementation of crowns and bridges onto implant abutments
HC	Provicol—VOCO (calcium hydroxide)	25 g base paste 25 g catalyst paste	VOCO	Temporary cementation of inlays, onlays, and crowns Partials, bridges, engagers, and pins Temporary restoration of small cavities

**Table 2:** Mean values of elastic modulus (gigapascal) and Poisson's ratio (*ν*).

**Property**	**Immediate**	**Aged**
*V*	0.8	0.48
*E* (GPa)	4.0	2.96

**Table 3:** Mean pull-out retention force and Tukey's grouping (95%).

**Property**	**Cement**	**Average (** **N** ** )**	**Bond strength (MPa)**	**Grouping**
Immediate	IOV	163.85 ± 29.86	2.55	A
	HC	122.34 ± 13.06	1.81	B
	OZnE	109.35 ± 20.10	1.62	B

Aged	IOV	46.89 ± 14.43	0.70	C
	HC	51.04 ± 21.79	0.80	C
	OZnE	48.48 ± 5.45	0.75	C

## Data Availability

Data is available on request from the first author.
